# The Book World of Medicine and Science

**Published:** 1903-11-14

**Authors:** 


### The Book World of Medicine and Science.

Clinical Treatises on the Pathology and Therapy of Disorders of Metabolism and Nutrition. By Dr. Carl von Noorden, Senior Physician to the City Hospital, Frankfurt. Authorised American Edition. Translated under the direction of Boardman Reed, M.D. Part I, Obesity. Part II, Nephritis. Part III., Membranous Catarrh of the Intestines. (New York: E. B. Treat & Co. 1903.)

Professor von Noorden's researches into derangements of nutritional processes are of such importance that we congratulate Dr. Boardman Reed on having undertaken the pleasurable duty of presenting them to English-reading students. The three monographs now before us are valuable contributions to the subjects with which they deal, and will prove not only suggestive in indicating means for further research but of real service in directing treatment along rational lines. Practitioners will find these volumes both scientific and practical. They are based upon experiments and bedside observations carried on under the direction of one who is eminent both as a pathologist and as a clinician. The translation has been well done, and successfully renders the author's vigorous and virile German into smooth and idiomatic English while preserving the spirit of the original. In the monograph on obesity a good review is given of the indications for "reduction cures." It is contended that such, provided it be really indicated and provided also the proper method is adopted and carried out expeditiously, is by no means a weakening procedure, particularly if the peculiarities of each individual case are carefully considered. So-called "sanitarium treatment" is recommended. The indications for the treatment of obesity complicated with diseases of the circulatory system, renal affections, chronic pulmonary lesions, chronic rheumatism, gout and derangements of the nervous system are well expressed and should prove of much service. The volume dealing with nephritis is of particular interest, for here the author boldly attacks much in customary procedure. He does not believe that light meats are to be considered safer than dark ones and seeks to show that the old view that milk is the best diet in all cases of nephritis is by no means correct. It is also contended that in many instances the ingestion of fluids needs to be restricted rather than encouraged. Dietetic therapy has for long been considered a most important matter in the management of renal cases, and practitioners would do well to revise their creed and study their practice in the light of this suggestive and we must add somewhat heterodox monograph. In the essay on colica mucosa the pathology of this obscure complaint is well discussed, its symptomatology described and full details as to its treatment are given. A useful bibliography is appended. These attractive studies should do much to quicken research into the many problems which still surround diseases of metabolism and nutrition, and will undoubtedly further the scientific treatment of some of these obscure affections.

Essentials of Pelvic Diagnosis. By E. Stanmore Bishop, F.R.C.S. (Bristol: John Wright and Co. 1903. Price 9s. 6d. Pp. 297. Illustrations 33. Plates 3.)

This is a truly remarkable book. The author himself seems to feel in his preface that he has had great temerity in bringing it out, but he fails to prove, as far as we can judge, that it will have much or any real value. The work starts with a clear account of the various methods of pelvic diagnosis, and the description and illustrations of the different positions into which the patient may be placed for pelvic examination in the female are distinctly good. The short account of the various instruments to be used in diagnosis does not leave much for improvement. But once this part of the book has been read, there begins a series of pages which are not only extremely difficult to follow, but tend to confuse. This part of the work is entitled "Lines of Diagnosis," and consists of a short description of the several diseases of the region of the pelvis, and even beyond the pelvis, and an attempt to state what such a review of the disease should call up into the mind of the clinician. We confess that we should not have considered that the external organs of generation were, strictly speaking, within the pelvis, or to be dealt with in a work discussing the diagnosis of pelvic diseases. Many of the terms which are used are either loose or inaccurate. The expression, for instance, "Swellings occurring midway between abdomen and scrotum," is hardly a satisfactory heading under which to place a spermatic varicocele, which by the way is said to affect both sexes. Again, under the designation of "swellings felt bi-manually," the author makes a "Group B.—In Males, or in both sexes," and under this heading diagnoses such lesions as enchondroma, psoas abscess, impacted ureteral calculus, proliferating ovarian cyst, congested ovary, etc., and we candidly confess that such a haphazard classification and differentiation is even more than nature herself demands of the observer! Part III. of the work is a series of "Diagnostic Tables," and here there is some room for congratulation upon the author's method. Although these tables are based on what has preceded them, yet they are more clearly written, and may be of some use to the student. But again it is somewhat startling to read such a heading as "Group B.—Common to both sexes. Condition distinct from Uterus." Lastly, we cannot look upon "tumours of pelvic origin, which have developed into the abdomen" as anything but careless writing. How can a pelvic tumour by any possibility "develop" into the abdomen? It may distend the abdomen, it may, that is to say by its increase in size, mount from out of the true pelvic cavity into that of the abdomen proper, but this is all. There are several misprints, and one in heavy type certainly requires correction. On page 81, "Scirrhus" is spelt "Schirrus." A series of "Illustrative Cases" are introduced in order to demonstrate how the somewhat complicated methods of the writer can be applied. The book ends with an excellent review by Dr. C. H. Melland of the modern-day methods of the examination of the blood in disease. The printing and general get up of the book are quite satisfactory. It is therefore with a considerable amount of regret that we cannot recommend this work for the purposes for which it was written.

